# Comparison of Estimated Glomerular Filtration Rate by the Chronic Kidney Disease Epidemiology Collaboration (CKD-EPI) Equations with and without Cystatin C for Predicting Clinical Outcomes in Elderly Women

**DOI:** 10.1371/journal.pone.0106734

**Published:** 2014-09-29

**Authors:** Wai H. Lim, Joshua R. Lewis, Germaine Wong, Robin M. Turner, Ee M. Lim, Peter L. Thompson, Richard L. Prince

**Affiliations:** 1 University of Western Australia School of Medicine and Pharmacology, Sir Charles Gairdner Hospital Unit, Perth, Australia; 2 Department of Renal Medicine, Sir Charles Gairdner Hospital, Perth, Australia; 3 Department of Endocrinology and Diabetes, Sir Charles Gairdner Hospital, Perth, Australia; 4 Centre for Kidney Research, Children's Hospital at Westmead, Sydney, Australia; 5 School of Public Health, Sydney Medical School, The University of Sydney, Sydney, Australia; 6 School of Public Health, The University of New South Wales, Sydney, Australia; 7 PathWest, Sir Charles Gairdner Hospital, Perth, Australia; 8 Department of Cardiovascular Medicine, Sir Charles Gairdner Hospital, Perth, Australia; University of São Paulo School of Medicine, Brazil

## Abstract

**Background:**

Reduced estimated glomerular filtration rate (eGFR) using the cystatin-C derived equations might be a better predictor of cardiovascular disease (CVD) mortality compared with the creatinine-derived equations, but this association remains unclear in elderly individuals.

**Aim:**

The aims of this study were to compare the predictive values of the Chronic Kidney Disease Epidemiology Collaboration (CKD-EPI)-creatinine, CKD-EPI-cystatin C and CKD-EPI-creatinine-cystatin C eGFR equations for all-cause mortality and CVD events (hospitalizations±mortality).

**Methods:**

Prospective cohort study of 1165 elderly women aged>70 years. Associations between eGFR and outcomes were examined using Cox regression analysis. Test accuracy of eGFR equations for predicting outcomes was examined using Receiver Operating Characteristic (ROC) analysis and net reclassification improvement (NRI).

**Results:**

Risk of all-cause mortality for every incremental reduction in eGFR determined using CKD-EPI-creatinine, CKD-EPI-cystatin C and the CKD-EPI-creatinine-cystatic C equations was similar. Areas under the ROC curves of CKD-EPI-creatinine, CKD-EPI-cystatin C and CKD-EPI-creatinine-cystatin C equations for all-cause mortality were 0.604 (95%CI 0.561–0.647), 0.606 (95%CI 0.563–0.649; p = 0.963) and 0.606 (95%CI 0.563–0.649; p = 0.894) respectively. For all-cause mortality, there was no improvement in the reclassification of eGFR categories using the CKD-EPI-cystatin C (NRI -4.1%; p = 0.401) and CKD-EPI-creatinine-cystatin C (NRI -1.2%; p = 0.748) compared with CKD-EPI-creatinine equation. Similar findings were observed for CVD events.

**Conclusion:**

eGFR derived from CKD-EPI cystatin C and CKD-EPI creatinine-cystatin C equations did not improve the accuracy or predictive ability for clinical events compared to CKD-EPI-creatinine equation in this cohort of elderly women.

## Introduction

Chronic kidney disease (CKD) is a major public health burden worldwide. Patients with CKD, especially those on dialysis, suffer from reduced life expectancy and quality of life [1]. CKD is a multi-system disease with established evidence demonstrating reduced kidney function increases the risk of cardiovascular disease (CVD) mortality [2]–[5], infections and cancer [6]. Previous meta-analyses reported the risk of associated disease such as CVD mortality commences with an estimated glomerular filtration rate (eGFR) of less than 60 ml/min/1.73 m^2^ and increases exponentially as one approaches end-stage renal disease (ESRD) requiring dialysis. However, epidemiological studies have also shown that eGFR between 60–74.9 mL/min/1.73 m^2^ is associated with a higher risk of CVD-related death compared to eGFR of ≥75 mL/min/1.73 m^2^ in patients following myocardial infarction suggesting that the risk of adverse clinical events is not confined to those with eGFR of less than 60 mL/min/1.73 m^2^ [7]. Although it is generally accepted that early identification of CKD may slow the progression to advanced stage kidney disease and provides a window of opportunity to prevent associated illness such as CVD and cancer [8], the threshold of reduced kidney function that prompts early intervention remains undefined suggesting that determining precise GFR in individuals may not be absolutely critical.

Chronic Kidney Disease Epidemiology Collaboration (CKD-EPI) equation [3] has been shown to be a more reliable marker of measured GFR and is superior in predicting the risk of adverse clinical outcomes such as mortality and stroke compared to Modification of Diet in Renal Disease (MDRD) [9] or the Cockcroft-Gault equations [10]. Although these equations are widely used in the community, previous studies have shown that serum creatinine-based equations may underestimate actual kidney function, especially in elderly individuals. As serum creatinine is affected by multiple factors including muscle mass and age, [11], alternative filtration markers such as cystatin C have been evaluated for GFR estimation.

Several newly-derived eGFR equations such as the CKD-EPI cystatin C and CKD-EPI creatinine-cystatin C equations have shown improvement in the precision and accuracy of determining GFR compared to CKD-EPI creatinine equation, but uncertainties remain as to the clinical significance and cost-effectiveness of using cystatin C-derived eGFR estimations over creatinine-derived eGFR estimations in the general population, particularly in elderly individuals. A recent meta-analysis of sixteen population cohorts reported both CKD-EPI cystatin C and combined CKD-EPI creatinine-cystatin C equations improved the accuracy in predicting all-cause and CVD mortality compared to CKD-EPI creatinine equation, but the majority of the included population cohorts were younger individuals of mixed gender with dissimilar proportion of muscle mass [12]. There have been no prior studies examining the clinical utility of these newly derived cystatin C equations in predicting adverse clinical outcomes exclusively in the older female population. The aims of this study were to determine the association of reduced kidney function as measured by CKD-EPI creatinine, CKD-EPI cystatin C and CKD-EPI creatinine-cystatin C equations and all-cause mortality and CVD events and also to assess the accuracy of these newly derived cystatin C-based eGFR equations in the prediction of clinical events in a cohort of elderly women mainly without prevalent CKD and with two-thirds of women with eGFR above 60 mL/min/1.73 m^2^.

## Subjects and Methods

### Study Population

One thousand five hundred women were recruited in 1998 to a five-year prospective, randomized, controlled trial of oral calcium supplements (1.2 g of elemental calcium daily or matching placebo) to prevent osteoporotic fractures, the Calcium Intake Fracture Outcome study (CAIFOS; Australian Clinical Trials Registry Registration Number: ACTRN012607000055404) [13]. Details of recruitment are published elsewhere [13]. Our population-based study is representative of the general elderly population in Western Australia. Participants were women aged over 70 years who were selected using the electoral roll and contacted by mail. Registration on this electoral roll is a standard and compulsory requirement of citizenship in Australia. Of the 5,586 women who responded to a letter inviting participation, 1510 eligible women were randomly selected. Participants had similar disease burden and pharmaceutical consumption to the whole population of this age but they were more likely to be from higher socio-economic groups [13]. The University of Western Australia Human Ethics Committee had approved the study and written informed consents were obtained from all participants. The present study is to evaluate the utility of creatinine and/or cystatin-derived eGFR equations in a cohort of elderly women recruited in 1998 in predicting 10-year clinical outcomes up to 2008.

Baseline medical history including the presence of diabetes, hypertension, smoking history (current/former smokers or non-smokers) and medications were obtained from all participants. Blood pressure was measured on the right arm with a mercury column manometer using an adult cuff after the participants have been seated in an upright position and had rested for 5 minutes. An average of three blood pressure readings was recorded.

Fasting blood samples were collected at baseline (i.e. at time of randomisation in 1998) with sera stored in −70°C freezer until analysis. Creatinine and cystatin C measurements were performed using stored sera after 2008 and results were available in 1165 women (77%). Serum creatinine was analysed using an isotope dilution mass spectrometry (IDMS) traceable Jaffe kinetic assay for creatinine on a Hitachi 917 analyser (Roche Diagnostics GmbH, Mannheim Germany). Serum cystatin C was measured on the Siemens Dade Behring Nephelometer, traceable to the International Federation of Clinical Chemistry Working Group for Standardization of Serum cystatin C and the Institute for Reference Materials and Measurements certified reference materials. eGFR was estimated by three equations derived by *Inker et al* and these are presented in Table S1 – CKD-EPI creatinine equation, CKD-EPI cystatin C equation and CKD-EPI creatinine-cystatin C equation [14].

### Assessment of clinical outcomes

Participants' general practitioners verified their medical histories and medications where possible, and were coded using the International Classification of Primary Care–Plus (ICPC-Plus) method [15]. Prevalent CVD was determined from hospital discharge data between 1980 and 1998 and were defined using diagnosis codes from the International Classification of Diseases, Injuries and Causes of Death Clinical Modification (ICD-9-CM, 309-459) [16]. Prevalent renal disease was collected between 1980 and 1998 using International Classification of Diseases, Injuries and Causes of Death Clinical Modification (ICD-9-CM) 17. These codes included glomerular diseases (ICD-9-CM codes 580–583); renal tubulo-interstitial diseases (ICD-9-CM codes 593.3–593.5, 593.7); renal failure (ICD-9-CM codes 584–586); and hypertensive renal disease (ICD-9-CM code 403). The search for renal disease hospitalizations included any diagnosis code.

The primary outcomes of the study were all-cause mortality and CVD hospitalizations and/or mortality retrieved from the Western Australian Data Linkage System (WADLS) for each of the study participants from 1998 until 10 years following their initial study visit. CVD hospitalizations and mortality were defined using primary diagnosis codes from ICD-9-CM, 390-459 [16] and the International Statistical Classification of Diseases and Related Health Problems, 10^th^ Revision, Australian Modification (ICD-10-AM), I00-I99 [17]. All diagnosis text fields from the death certificate were used to ascertain the cause(s) of deaths where these data were not yet available from the WADLS.

### Statistical Analysis

Baseline characteristics were expressed as mean and standard deviation (SD) for continuous variables or as number and proportion for categorical variables. Association between eGFR and all-cause mortality and CVD hospitalization and/or mortality was examined using Cox proportional hazard regression model and results were expressed as hazard ratio (HR) with 95% confidence interval (CI) for every incremental reduction in eGFR to allow comparison between equations. The covariates included in the Cox regression models were age, smoking history, body mass index (BMI), diabetes, antihypertensive medications, systolic blood pressure, treatment code, prevalent renal and CVD.

To assess performance of the different equations for estimating eGFR, we assessed the discrimination of the three different models using the Area Under Curve (AUC). Discrimination refers to how well the model distinguishes individuals with and without the outcomes of interests. To assess discrimination, we calculated the area under the receiver operating characteristic (ROC) curve (AUC). An area of 1 implies perfect discrimination, whereas an area of 0.5 represents random discrimination. The sidak option provides adjusted p-values comparing the ROC areas between eGFR equations, assuming a “gold standard” being the CKD-EPI creatinine equation. For net reclassification improvement (NRI), participants were classified into three eGFR categories for all-cause and CVD hospitalization and/or mortality (≥75, 60–74.9 and <60 mL/min/1.73 m^2^), and then reclassified into new eGFR categories with CKD-EPI cystatin C equation and CKD-EPI creatinine-cystatin C equation as compared with CKD-EPI creatinine equation. P-values of less than 0.05 in two tailed testing were considered statistically significant. The data was analysed using SPSS (version 15; SPSS Inc, Chicago, IL) and STATA (version 11 StataCorp LP, College Station, TX).

## Results

### Baseline characteristics

The baseline characteristics of study cohort as of 1998 are shown in table 1. The mean ± SD age of the participants was 75±2.7 years. Among them, 42.7% had hypertension, 6.7% had diabetes and 36.5% were former/current smokers at the inception of the study. Using hospital discharge records, 23.4% of participants were deemed to have prevalent CVD (defined as having prior hospitalizations for CVD) and 1.5% prevalent renal disease (defined as having prior hospitalizations of any renal disease) between 1980 to study randomization. The mean ± SD eGFRs calculated by CKD-EPI creatinine equation, CKD-EPI cystatin C equation and CKD-EPI creatinine-cystatin C equation were 66.6±13.3, 65.3±14.8 and 65.7±13.0 mL/min/1.73 m^2^ respectively.

**Table 1:** Baseline characteristics of the cohort.

Baseline Characteristics	All participants (n = 1,165)
**Age, mean ± SD, years**	75.2±2.7
**Body mass index, mean ± SD, kg/m^2^**	27.2±4.7
**Systolic blood pressure, mean ± SD, mmHg**	138.0±17.9
**Diastolic blood pressure, mean ± SD, mmHg**	73.1±11.0
**Anti-hypertensive medications, No. (%)**	497 (42.7)
**Smoked ever, No. (%)**	427 (36.5)
**Diabetes, No. (%)**	78 (6.7)
**Cardiovascular disease at baseline (I00-I99), No. (%)**	273 (23.4)
**Renal disease at baseline, No. (%)**	17 (1.5)
**Calcium supplements, No. (%)**	614 (52.7)
**Biochemistry**	
**Creatinine, mean ± SD, mg/dL**	0.9±0.2
**Cystatin C, mean ± SD, mg/L**	1.1±0.2
**Estimated glomerular filtration rate by the CKD-EPI equations**	
**CKD-EPI creatinine-derived eGFR, mean ± SD, mL/min/1.73 m^2^**	66.6±13.3
**CKD-EPI Cystatin C-derived eGFR, mean ± SD, mL/min/1.73 m^2^**	65.3±14.8
**CKD-EPI Creatinine-cystatin C-derived eGFR, mean ± SD, mL/min/1.73 m^2^**	65.7±13.0

Results are mean ± SD or number and (%). CVD cardiovascular disease, eGFR estimated glomerular filtration rate, CKD-EPI Chronic Kidney Disease EPIdemiology.

### Association between eGFR, cardiovascular events and all-cause mortality

There was at least over 30% increase in CVD events between participants with eGFR of <60 mL/min/1.73 m^2^ compared to those with eGFR of ≥75 mL/min/1.73 m^2^ as measured by the CKD-EPI creatinine, CKD-EPI cystatin C and the CKD-EPI creatinine-cystatic C equations (Table 2). However, there was no association between eGFR reduction and all-cause mortality for the three eGFR equations (Table 2). For CKD-EPI creatinine equation, the proportion of participants with prevalent renal disease in those with eGFR of <60, 60–75 and>75/mL/min/1.73 m^2^ were 0.9%, 0.6% and 1.9% respectively (χ^2^ 3.01, p = 0.222), which was similar for CKD-EPI cystatin C and CKD-EPI creatinine-cystatin C equations.

**Table 2:** Hazard ratios for clinical outcomes stratified by categories of eGFR in ml/min/1.73 m^2^.

Characteristics (n = 1,165)	Hazard Ratio (95% CI)	P value
**All-cause mortality (n = 231)**		
**CKD-EPI creatinine equation**		0.318
≥75 ml/min/1.73 m^2^	Referent	
60–74.9 ml/min/1.73 m^2^	0.89 (0.63–1.26)	
<60 ml/min/1.73 m^2^	1.14 (0.81–1.60)	
**CKD-EPI cystatin C equation**		0.208
≥75 ml/min/1.73 m^2^	Referent	
60–74.9 ml/min/1.73 m^2^	0.76 (0.53–1.07)	
<60 ml/min/1.73 m^2^	0.96 (0.68–1.35)	
**CKD-EPI creatinine-cystatin C equation**		0.278
≥75 ml/min/1.73 m^2^	Referent	
60–74.9 ml/min/1.73 m^2^	0.80 (0.56–1.14)	
<60 ml/min/1.73 m^2^	1.01 (0.71–1.45)	
**Cardiovascular disease hospitalization/mortality (n = 469)**		
**CKD-EPI creatinine equation**		0.028
≥75 ml/min/1.73 m^2^	Referent	
60–74.9 ml/min/1.73 m^2^	1.08 (0.84–1.39)	
<60 ml/min/1.73 m^2^	1.36 (1.06–1.76)	
**CKD-EPI cystatin C equation**		0.031
≥75 ml/min/1.73 m^2^	Referent	
60–74.9 ml/min/1.73 m^2^	1.06 (0.81–1.37)	
<60 ml/min/1.73 m^2^	1.35 (1.04–1.75)	
**CKD-EPI creatinine-cystatin C equation**		0.021
≥75 ml/min/1.73 m^2^	Referent	
60–74.9 ml/min/1.73 m^2^	1.21 (0.92–1.57)	
<60 ml/min/1.73 m^2^	1.46 (1.11–1.91)	

Results are age and multivariable-adjusted hazard ratio (mean 95% CI) by eGFR categories by the three equations. eGFR - estimated glomerular filtration rate, CKD-EPI - Chronic Kidney Disease EPIdemiology equation. Multivariable adjustment includes age, body mass index, previous cardiovascular disease, previous renal disease, systolic blood pressure, antihypertensive medications, diabetes, smoking history and treatment.

### Model discrimination

**Figure 1:**
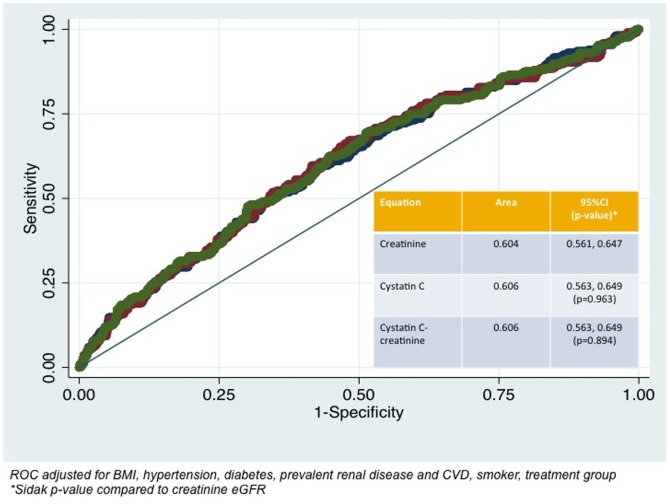
Receiver Operating Characteristic curves of CKD-EPI creatinine, CKD-EPI cystatin C and CKD-EPI creatinine-cystatin C eGFR equations for all-cause mortality. Fully adjusted models include body mass index, previous cardiovascular disease, previous renal disease, anti-hypertensive medications, diabetes, smoking history and treatment code.

**Figure 2:**
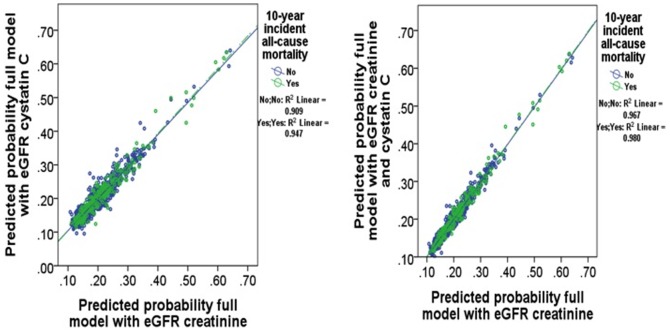
Receiver Operating Characteristic curves of CKD-EPI creatinine, CKD-EPI cystatin C and CKD-EPI creatinine-cystatin C eGFR equations for cardiovascular disease hospitalization and/or mortality. Fully adjusted models include body mass index, previous cardiovascular disease, previous renal disease, anti-hypertensive medications, diabetes, smoking history and treatment code.

For prediction of all-cause mortality, the AUCs varied between 0.604 (95%CI 0.561, 0.647), 0.606 (95%CI 0.563, 0.649; Sidak p-value 0.963) and 0.606 (95%CI 0.563, 0.649; Sidak p-value 0.894) respectively using the CKD-EPI creatinine, CKD-EPI cystatin C and CKD-EPI creatinine-cystatin C equations adjusted for age, BMI, hypertension, diabetes, systolic blood pressure, prevalent renal disease and CVD, smoking history and treatment group (Figure 1). The correlation between the predicted probabilities of the adjusted model for all cause mortality using CKD-EPI creatinine equation compared with CKD-EPI creatinine-cystatin C equation is shown in Figure 2.

**Figure 3:**
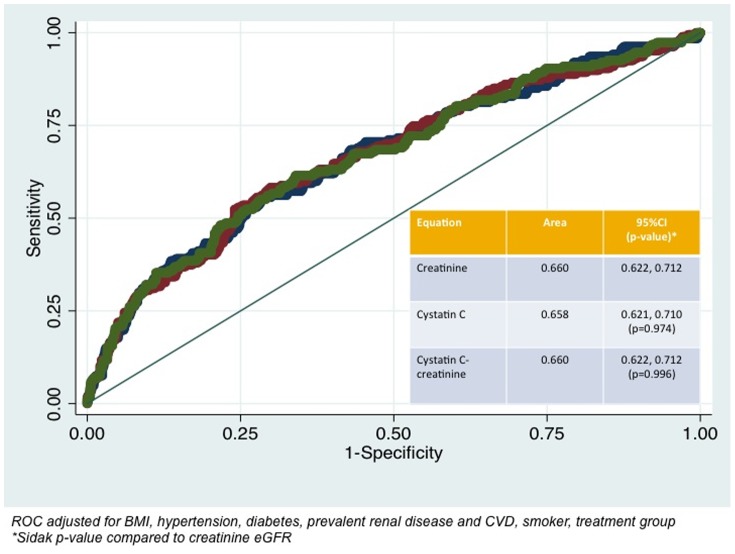
Correlation of the predicted probabilities for cardiovascular disease hospitalization and/or mortality of CKD-EPI creatinine and CKD-EPI creatinine-cystatin C equations. Fully adjusted models include body mass index, previous cardiovascular disease, previous renal disease, anti-hypertensive medications, diabetes, smoking history and treatment code.

**Figure 4:**
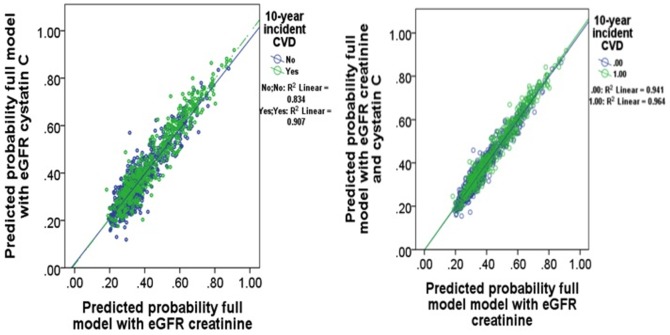
Correlation of the predicted probabilities for all-cause mortality of CKD-EPI creatinine and CKD-EPI creatinine-cystatin C equations. Fully adjusted models include body mass index, previous cardiovascular disease, previous renal disease, anti-hypertensive medications, diabetes, smoking history and treatment code.

For the prediction of CVD hospitalization and/or mortality, the AUCs varied between 0.660 (95%CI 0.622, 0.712), 0.659 (95%CI 0.621, 0.710; Sidak p-value 0.974) and 0.660 (95%CI 0.622, 0.712; Sidak p-value 0.996) respectively using the CKD-EPI creatinine, CKD-EPI cystatin C and CKD-EPI creatinine-cystatin C equations adjusted for age, BMI, hypertension, diabetes, systolic blood pressure, prevalent renal disease and CVD, smoking history and treatment group (Figure 3). The correlation between the predicted probabilities of the adjusted model for CVD hospitalization and/or mortality using CKD-EPI creatinine equation compared with CKD-EPI creatinine-cystatin C equation is shown in Figure 4.

### Net reclassification improvement

The reclassification of eGFR categories in predicting all-cause mortality and CVD hospitalization and/or mortality between CKD-EPI cystatin C and CKD-EPI creatinine-cystatin C equations compared with CKD-EPI creatinine equation is shown in Tables 3 and 4. For all-cause mortality, there was no significant improvement in net reclassification of eGFR categories with CKD-EPI cystatin C equation (NRI -4.1%, p = 0.401) or CKD-EPI creatinine-cystatin C equation (NRI -1.2%, p = 0.748) compared with CKD-EPI creatinine equation. For CVD hospitalization and/or mortality, there was no significant improvement in net reclassification of eGFR categories for CVD hospitalization and/or mortality with CKD-EPI cystatin C equation (NRI 2.0%, p = 0. 614) and creatinine-cystatin C equation (NRI 3.0%, p = 0.351) compared with CKD-EPI creatinine equation.

**Table 3:** Net reclassification improvement of eGFR categories for all-cause mortality and cardiovascular disease hospitalization and/or mortality using CKD-EPI cystatin C equation compared with CKD-EPI creatinine equation.

All-cause mortality (Net reclassification improvement -4.1%, p = 0.401)						
	**eGFR with CKD-EPI cystatin C equation**					
**eGFR with CKD-EPI creatinine equation**	≥75	60–74.9	<60	Reclassified higher eGFR	Reclassified lower eGFR	Correctly reclassified
	**Participants who died (n = 231)**					
≥75	32	19	9	48 (20.8%)	50 (21.6%)	2 (0.8%)
60–74.9	22	37	22			
<60	6	20	64			
	**Participants who did not die (n = 934)**					
≥75	124	105	38	195 (20.9%)	241 (25.8%)	46 (4.9%)
60–74.9	95	191	98			
<60	20	80	183			

Cardiovascular disease hospitalization and/or mortality (Net reclassification improvement 2.0%, p = 0.614)						
	**eGFR with CKD-EPI cystatin C equation**					
**eGFR with CKD-EPI creatinine equation**	≥75	60–74.9	<60	Reclassified higher eGFR	Reclassified lower eGFR	Correctly reclassified
	**Participants with an event (n = 469)**					
≥75	50	42	18	91 (19.4%)	116 (24.7%)	25 (5.3%)
60–74.9	37	83	56			
<60	9	45	129			
	**Participants without an event (n = 696)**					
≥75	106	82	29	152 (21.8%)	175 (25.1%)	23 (3.3%)
60–74.9	80	145	64			
<60	17	55	118			

CVD risk factors include age, body mass index, previous cardiovascular disease, previous renal disease, systolic blood pressure, anti-hypertensive medications, diabetes, smoking history and treatment code. CVD indicates cardiovascular disease; eGFR estimated glomerular filtration rate and CKD-EPI - Chronic Kidney Disease Epidemiology equation.

**Table 4:** Net reclassification improvement of eGFR categories for all-cause mortality and cardiovascular disease hospitalization and/or mortality using CKD-EPI creatinine-cystatin C equation compared with CKD-EPI creatinine equation.

All-cause mortality (Net reclassification improvement -1.2%, p = 0.748)						
	**eGFR with CKD-EPI creatinine-cystatin C equation**					
**eGFR with CKD-EPI creatinine equation**	≥75	60–74.9	<60	Reclassified higher eGFR	Reclassified lower eGFR	Correctly reclassified
	**Participants who died (n = 231)**					
≥75	41	19	0	30 (13.0%)	36 (15.6%)	6 (2.6%)
60–74.9	13	51	17			
<60	0	17	73			
	**Participants who did not die (n = 934)**					
≥75	178	85	4	110 (11.8%)	146 (15.6%)	36 (3.8%)
60–74.9	52	275	57			
<60	0	58	225			

Cardiovascular disease hospitalization and/or mortality (Net reclassification improvement 3.0%, p = 0.351)						
	**eGFR with CKD-EPI creatinine-cystatin C equation**					
**eGFR with CKD-EPI creatinine equation**	≥75	60–74.9	<60	Reclassified higher eGFR	Reclassified lower eGFR	Correctly reclassified
	**Participants with an event (n = 469)**					
≥75	70	38	2	47 (10.0%)	72 (15.4%)	25 (5.4%)
60–74.9	16	128	32			
<60	0	31	152			
	**Participants without an event (n = 696)**					
≥75	149	66	2	93 (13.4%)	110 (15.8%)	17 (2.4%)
60–74.9	49	198	42			
<60	0	44	146			

CVD risk factors include age, body mass index, previous cardiovascular disease, previous renal disease, systolic blood pressure, anti-hypertensive medications, diabetes, smoking history and treatment code. CVD indicates cardiovascular disease; eGFR estimated glomerular filtration rate and CKD-EPI - Chronic Kidney Disease Epidemiology equation.

## Discussion

In elderly individuals, the accurate evaluation of eGFR for CKD staging is critical to determine correct drug dosing and risk stratification for major clinical events including CVD and all-cause mortality. Our study findings suggest that the association between reduced GFR and clinical outcomes is similar for eGFR equations with and without cystatin C. In addition, the combined CKD-EPI creatinine-cystatin C eGFR or CKD-EPI cystatin C prediction equations were not superior in predicting or reclassifying CVD hospitalization and/or mortality or all-cause mortality over the CKD-EPI creatinine eGFR equation in a cohort of elderly women.

Cystatin C appears to be a superior GFR marker compared to creatinine [18], [19]. Cystatin C is a low molecular weight protein (13 kDa) that is produced at a constant rate by all cells in the body, is freely filtered by the glomeruli and is completely reabsorbed and catabolised by the proximal tubules. Unlike creatinine, cystatin C is less likely to be influenced by muscle mass or diet and therefore may be a more reliable marker of GFR, particularly in older individuals, females and those with reduced muscle mass [20]. In several studies, compared with creatinine, cystatin C is more accurate in stratifying the risk of CVD and all-cause mortality in elderly individuals [21]. In a cohort of 3,075 participants aged over 70 years, each SD reduction (0.3 g/L) in cystatin C concentration was associated with an increased risk of all-cause mortality (HR 1.24, 95% CI 1.20, 1.28) and CVD mortality (HR 1.20, 95% CI 1.11, 1.30) [22]. In a population-based prospective observational cohort of 9988 individuals aged 45–64 years, cystatin C level was a much stronger predictor of all-cause mortality, coronary artery disease events, heart failure events and end-stage renal disease compared to estimates of GFR derived from CKD-EPI creatinine equation [23]. Other studies have corroborated these findings and have also shown that cystatin C level may identify the group of CKD patients that may not be identified by CKD-EPI equation as being at high risk of CVD events and all-cause mortality [24], [25]. In contrast, a recent study by *Eriksen et al.* has shown that cystatin C was not superior in estimating measured GFR compared to creatinine in the general population [26] and other studies have suggested that the strong association between cystatin C and CVD or all-cause mortality may be related to other factors including body size and the presence of diabetes and inflammation [27]. The discrepant findings between studies may reflect dissimilar population of varying ages, differences in participants' characteristics such as BMI and presence of comorbidities.

Two recently developed CKD-EPI creatinine-cystatin C and CKD-EPI cystatin C equations were shown to perform better in predicting measured radionuclide GFR compared to CKD-EPI creatinine equation [14]. Although bias was similar in all three eGFR equations in predicting measured GFR, the combined CKD-EPI creatinine-cystatin C equation had greater precision and accuracy resulting in a more accurate classification of measured GFR as <60 ml/min/1.73 m^2^. The use of the combined CKD-EPI creatinine-cystatin C equation was able to improve reclassification of individuals with creatinine-derived eGFR of 45–74 ml/min/1.73 m^2^ (net reclassification index 19.4; 95% CI, 8.7 to 30.1; P<0.001), and also 17% of individuals with creatinine-based eGFR of 45–59 ml/min/1.73 m^2^ to ≥60 ml/min/1.73 m^2^. In a recent meta-analysis of 11 general population studies comprising of 90,750 participants, there was a more consistent linear association between reduced eGFR derived from CKD-EPI cystatin C and CKD-EPI creatinine-cystatin C equations and increased risks of all-cause and CVD mortality for all eGFR values below 85 mL/min/1.73 m^2^ compared with CKD-EPI creatinine equation, well above the threshold of 60 mL/min/1.73 m^2^ for the detection of CKD with CKD-EPI creatinine-based eGFR [12]. The NRI using either CKD-EPI cystatin C-derived equations for all-cause and CVD mortality was 0.23 (95%CI 0.18, 0.28) and 0.17 (95%CI 0.11, 0.23) respectively suggesting that cystatin C-derived eGFR equations strengthens the association between eGFR and clinical outcomes. However, in this meta-analysis, there were only two studies that have included exclusively elderly participants with mean age of over 70 years. The ULSAM study from Sweden included only men and the CHS study included 41% men and 17% participants were of Black race [28], [29]. In both these studies, there was a large difference in mean eGFR across the three equations, with eGFR derived from CKD-EPI creatinine equation being much higher compared to both cystatin C equations. Our study has shown that the newly derived CKD-EPI cystatin C and CKD-EPI creatinine-cystatin C equations did not improve reclassification of eGFR categories that predicted the risk of CVD hospitalization and/or mortality or all-cause mortality compared to the commonly used CKD-EPI creatinine equation. The observed differences to the result of the meta-analysis may reflect dissimilar population characteristics with the studies included in the meta-analysis comprising men and women across all age categories and ethnicity compared to only elderly Caucasian women in our study. In addition, all elderly participants in this study were relatively healthy over the age of 70 with mild renal dysfunction, with the majority of participants within a relatively narrow range of eGFRs. In the two population cohorts of similar age (CHS and ULSAM studies), there were major differences in gender, race, BMI, comorbid status and baseline creatinine compared to this cohort, which may have contributed to the differences in study findings. There may also be potential errors in creatinine and cystatin C measurements and insufficient power in our study to detect significant differences between the eGFR equations or to detect a significant associations between these equations and all-cause mortality, which all may have contributed to differences in the reported study findings.

The strengths of this study include the use of a large prospective cohort of subjects with complete and accurate data collection over a 10-year period. We were able to accurately examine the association between estimates of GFR using the newly developed cystatin C equations and clinical outcomes in a population with a low prevalence of CVD and renal diseases, which further strengthens this association. However, the strengths of the study must be balanced against the limitations, which include a lack of radionuclide GFR measurements and availability of single time-point measurements of creatinine and cystatin C to estimate baseline GFR. In addition, our study cohort only included white female participants with presumed adequate nutrition and muscle mass (BMI mean ± SD of 27±5 kg/m^2^) and therefore the applicability of our study findings to males, other ethnic minorities or racial groups and those with poor nutrition and low muscle mass remains unclear.

In conclusion, the newly developed CKD-EPI cystatin C and combined CKD-EPI creatinine-cystatin C-derived eGFR equations were not superior in predicting CVD events or all-cause mortality compared with the commonly used CKD-EPI creatinine-derived eGFR equation in older female subjects with no or early CKD and this data cannot be extrapolated to older individuals with more advanced CKD. With a substantial cost-difference between measurements of creatinine and cystatin C, together with the uncertainty of the value of cystatin C-derived eGFR equations in predicting clinical events over creatinine-derived eGFR equation, the utility and cost-effectiveness of cystatin C in the elderly must be investigated further prior to implementation in clinical practice.

## Supporting Information

Table S1 **CKD-EPI equations using creatinine or cystatin C or a combination of creatinine and cystatin C.**
(TIF)
