# Supplementary figures and images for: Comparison of Estimated Glomerular Filtration Rate by the Chronic Kidney Disease Epidemiology Collaboration (CKD-EPI) Equations with and without Cystatin C for Predicting Clinical Outcomes in Elderly Women
Source: PLoS One. 2014 Sep 29;9(9):e106734. doi: 10.1371/journal.pone.0106734 (PMC4180254; doi:10.1371/journal.pone.0106734)

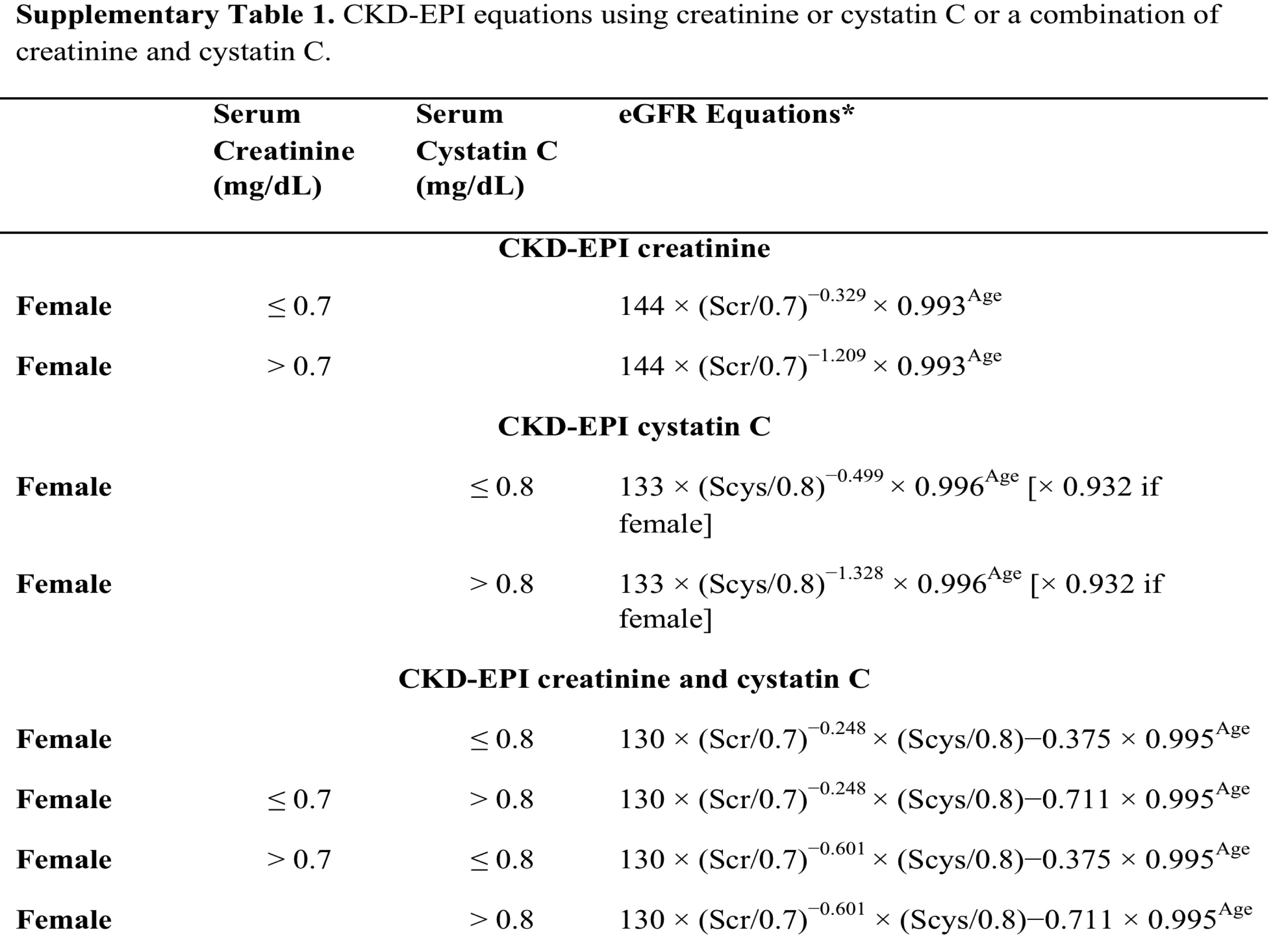

Supplement: Table S1 — CKD-EPI equations using creatinine or cystatin C or a combination of creatinine and cystatin C. (TIF) [file pone.0106734.s001.tif]
